# Supplementary material for: CDK4/6 inhibitors target SMARCA4-determined cyclin D1 deficiency in hypercalcemic small cell carcinoma of the ovary
Source: Nat Commun. 2019 Feb 4;10:558. doi: 10.1038/s41467-018-06958-9 (PMC6361890; doi:10.1038/s41467-018-06958-9)
Supplement: Supplementary file 1 — Supplementary Information [file 41467_2018_6958_MOESM1_ESM.pdf]

Supplementary Information

CDK4/6 inhibitors target SMARCA4-determined cyclin D1 deficiency in hypercalcemic small cell carcinoma of the ovary

Xue et al

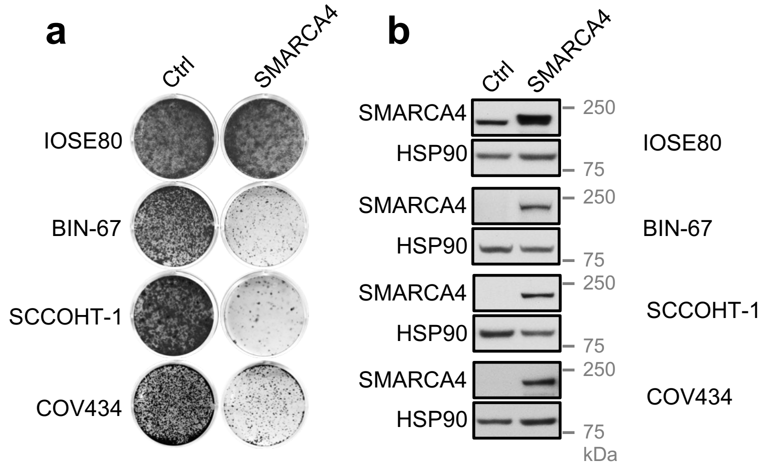

Supplementary Figure 1 | SMARCA4 Restoration in SCCOHT cells results in strong growth inhibition.

a, Forced SMARCA4 expression results in strong growth inhibition in SCCOHT cells (BIN-67, SCCOHT-1 and COV434) but not in the non-transformed ovarian epithelial control cells (IOSE80). Equal number of cells expressing lentiviral pReceiver vector control or pReceiver -*SMARCA4* were seeded after 2 days of selection for stable integration and then cultured for 15 days. For each cell line, all dishes were fixed at the same time, stained and photographed.

b, Western blot analysis of SMARCA4 expression in the cells described above. HSP90 was used as a loading control.

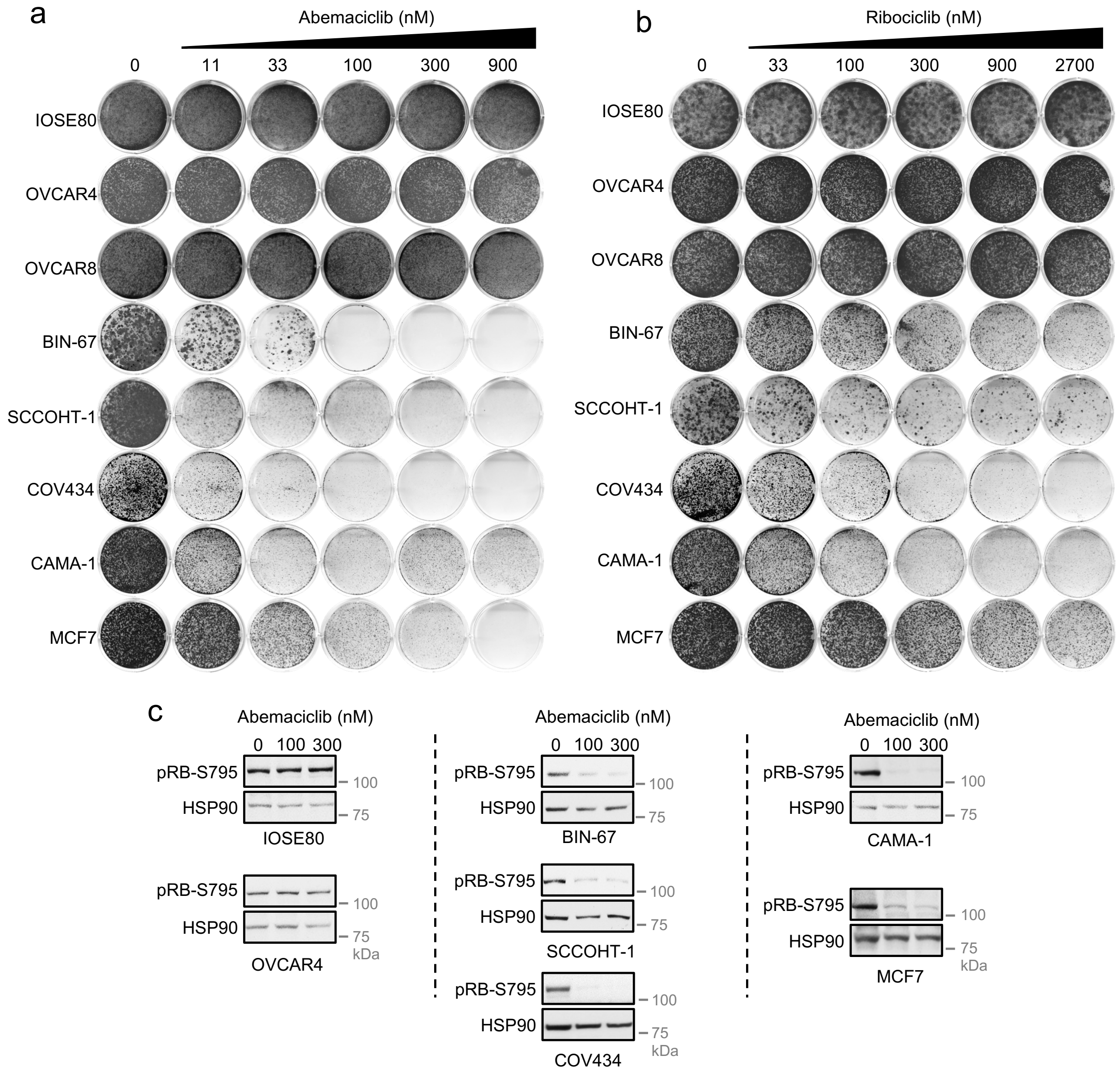

Supplementary Figure 2 | SCCOHT cells are highly sensitive to additional CDK4/6 inhibitors abemaciclib and ribociclib.

a, b, BIN-67, SCCOHT-1 and COV434 cells but not SMARCA4-proficient IOSE80 and ovarian carcinoma OVCAR4 and OVCAR8 cells are highly sensitive to abemaciclib and ribociclib, similar to ER^+ breast cancer cells MCF7 and CAMA-1. Colony formation assays of above cell lines cultured in the absence or presence of abemaciclib (**a**) and ribociclib (**b**) at the indicated concentrations for 10-25 days. For each cell line, all dishes were fixed at the same time, stained and photographed.

c, Abemaciclib treatment suppresses RB phosphorylation in both SCCOHT (BIN-67, SCCOHT-1, COV434) and ER+ breast cancer cells (CAMA-1 and MCF7), but not in IOSE80 and OVCAR4 cells. Levels of pRB-S795 in cells treated with 0 nM, 100nM or 300nM of abemaciclib for 24h were documented by western blot analysis. HSP90 was used as a loading control.

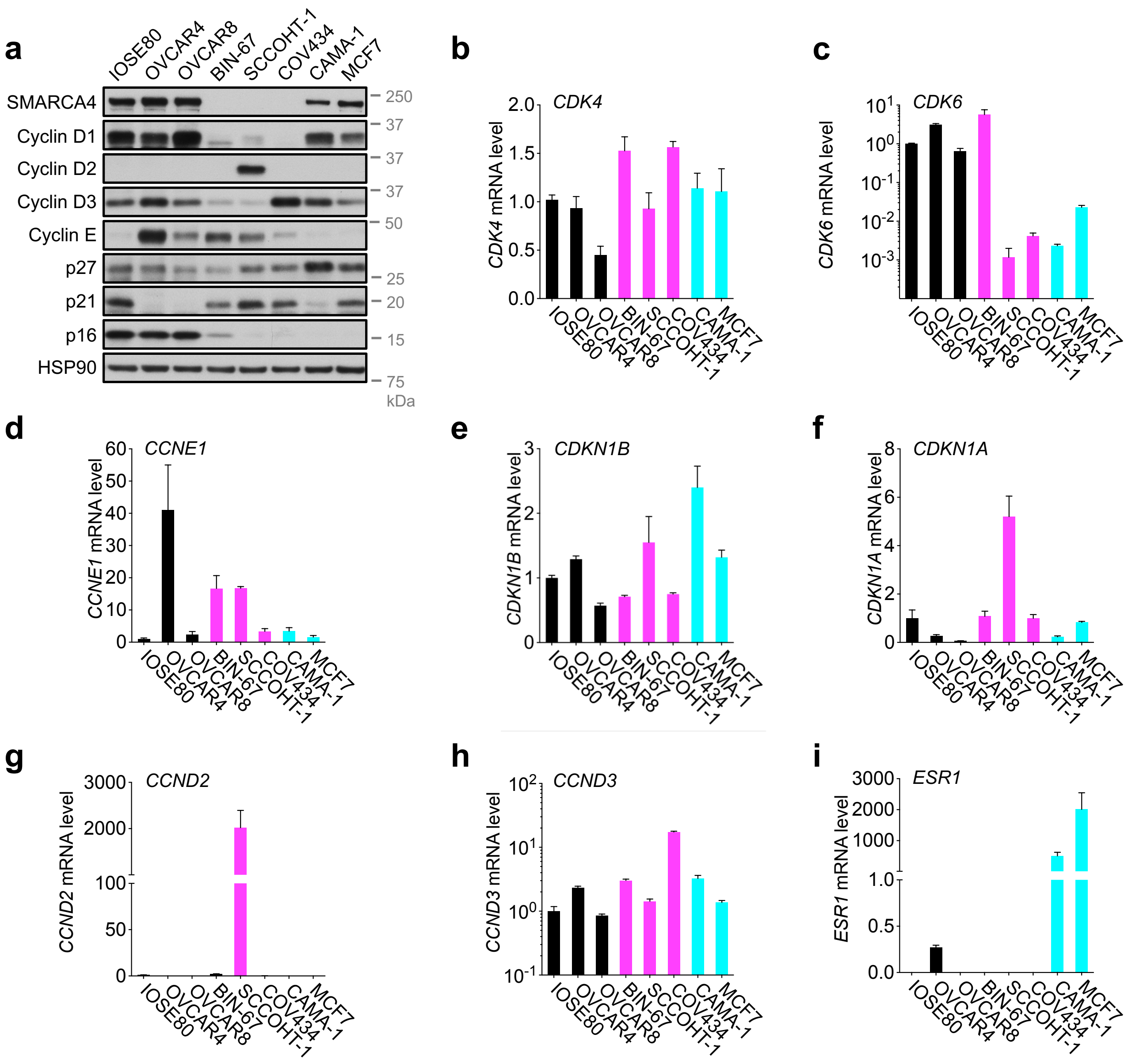

Supplementary Figure 3 | Expression of relevant cell cycle genes in SCCOHT and control cells.

a, Western blot analysis of SMARCA4, cyclin D1, cyclin D2, cyclin D3, cyclin E, p27, p21 and p16 in in a cell line panel consisting of non-transformed epithelial ovarian (IOSE80), ovarian carcinoma (OVCAR4, OVCAR8), SCCOHT (BIN-67, SCCOHT-1, COV434) and ER^{+} breast cancer (CAMA-1, MCF7) cells. HSP90 served as a loading control.

b-i, Relative mRNA levels of indicated genes (normalized to *GAPDH*) in the above cell line panel were measured by qRT-PCR. *CDK4*(**b**), *CDK6* (**c**), *CCNE1* (**d**), *CDKN1B* (**e**), *CDKN1A* (**f**), *CCND2* (**g**), *CCND3* (**h**) & *ESR1* (**i**) expression. Error bars: mean ± s.d. of biological replicates (n=3). SCCOHT cells: magenta; breast cancer cells: turquoise.

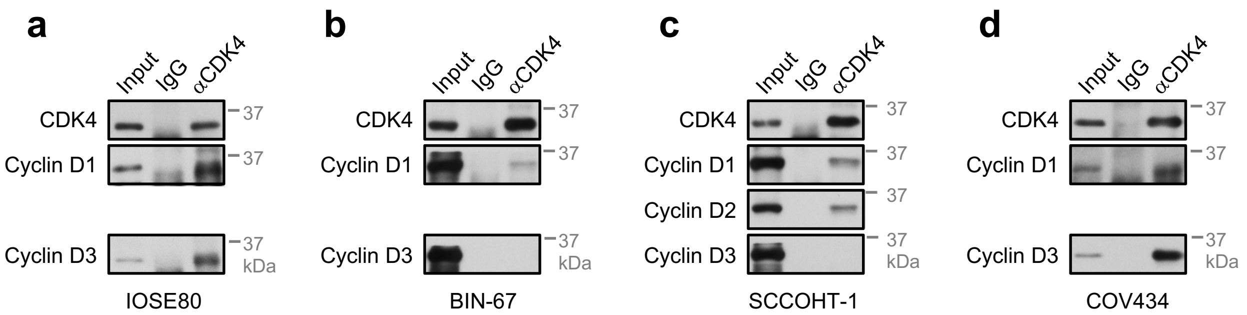

Supplementary Figure 4 | D type cyclins form complex with CDK4 in SCCOHT and SMARCA4-proficient cells.

a-d, CDK4/cyclin D complexes in the SMARCA4-proficient IOSE80 control and SCCOHT (BIN-67, SCCOHT-1, COV434) cells. CDK4 immunoprecipitations were performed using an antibody against CDK4 or IgG control and followed by western blotting of CDK4, cyclin D1, cyclin D2 and cyclin D3.

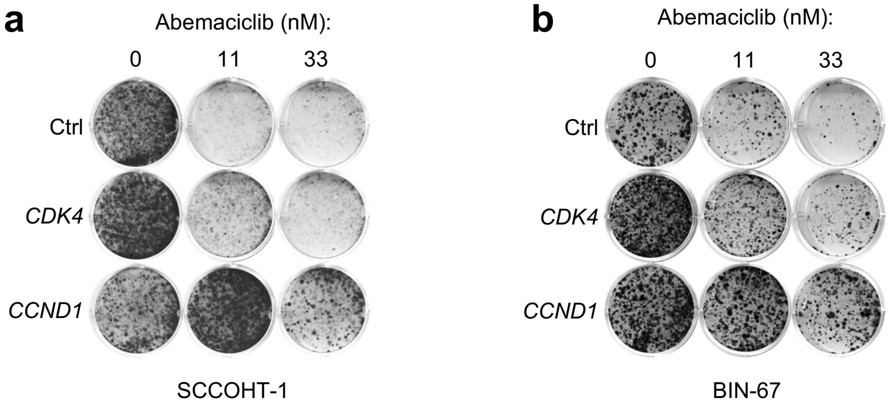

Supplementary Figure 5 | Ectopic expression of cyclin D1 but not CDK4 confers resistance to abemaciclib in SCCOHT-1 and BIN-67 cells.

Colony formation assay of SCCOHT-1(a) and BIN-67 (b) cells stably expressing control vectors (pLX304-*GFP* + pLX317-*GFP*), *CDK4* (pLX304-*GFP* + pLX317-*CDK4*) and *CCND1* (pLX304-*CCND1* + pLX317-*GFP*) treated with increasing concentration of abemaciclib. Same cells were used as in Fig. 3 h and i.

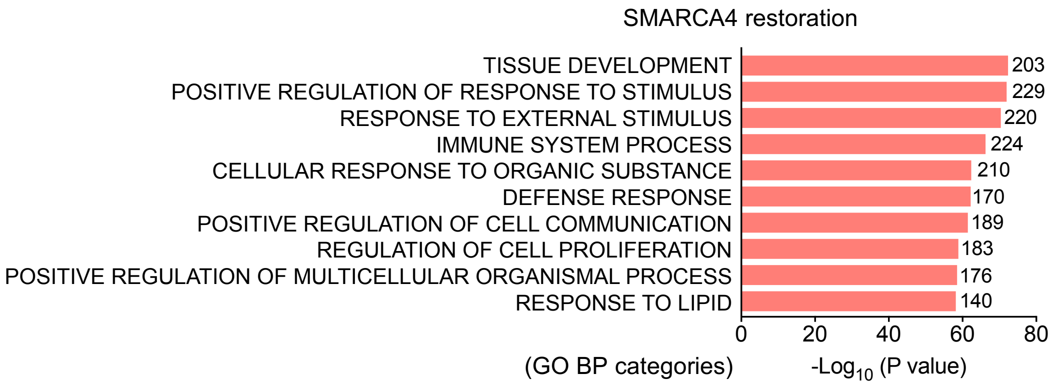

Supplementary Figure 6 | Top 10 gene signatures affected by SMARCA4 restoration in BIN-67 and SCCOHT-1 cells

RNA-Seq was performed in BIN-67 and SCCOHT-1 cells with SMARCA4 restoration in triplicates. Common genes that significantly changed (fold change>3, adjusted p <0.05) in both cell lines were analysed with GSEA. Top ten cellular processes by GO term are shown.

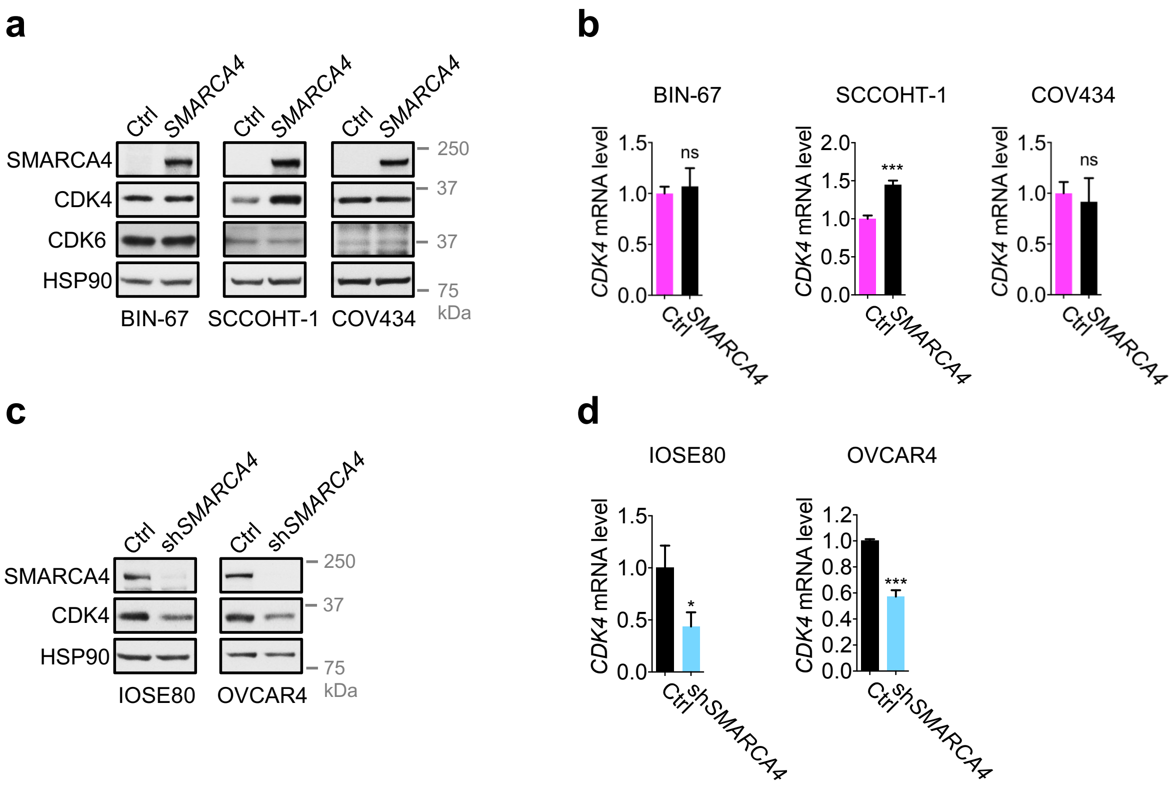

Supplementary Figure 7 | Role of SMARCA4 on CDK4 expression in SCCOHT and control cells

a, b, Restoration of SMARCA4 upregulated CDK4 protein (**a**) and *CDK4* mRNA (**b**) levels in SCCOHT-1 but not BIN-67 and COV434 cells. BIN-67, SCCOHT-1 and COV434 cells expressing lentiviral pReceiver vector control or pReceiver-*SMARCA4* were selected for 2 days for stable integration and then harvested for RNA and protein lysates. **a**, Western blot analysis for the indicated proteins in the cells described above. HSP90 served as loading control. **b**, Relative expression levels of *CDK4* mRNA (normalized to *GAPDH*) in these cells were measured by qRT-PCR. Error bars: mean ± s.d. of biological replicates (n=3; two-tailed *t*-test, *** $p < 0.001$). ; ns, not significant.

c, **d**, SMARCA4 knockdown in control cells suppressed CDK4 protein (c) and *CDK4* mRNA (d) levels. IOSE80 and OVCAR4 cells expressing lentiviral pLKO vector control or shRNA targeting SMARCA4 were selected for 2 days for stable integration and then harvested for RNA and protein lysates. **c**, Western blot analysis for the indicated proteins in the cells described above. HSP90 served as loading control. **d**, Relative expression levels of *CDK4* mRNA (normalized to *GAPDH*) in these cells were measured by qRT-PCR. Error bars: mean ± s.d. of biological replicates (n=3; two-tailed *t*-test, * $p < 0.05$, *** $p < 0.001$).

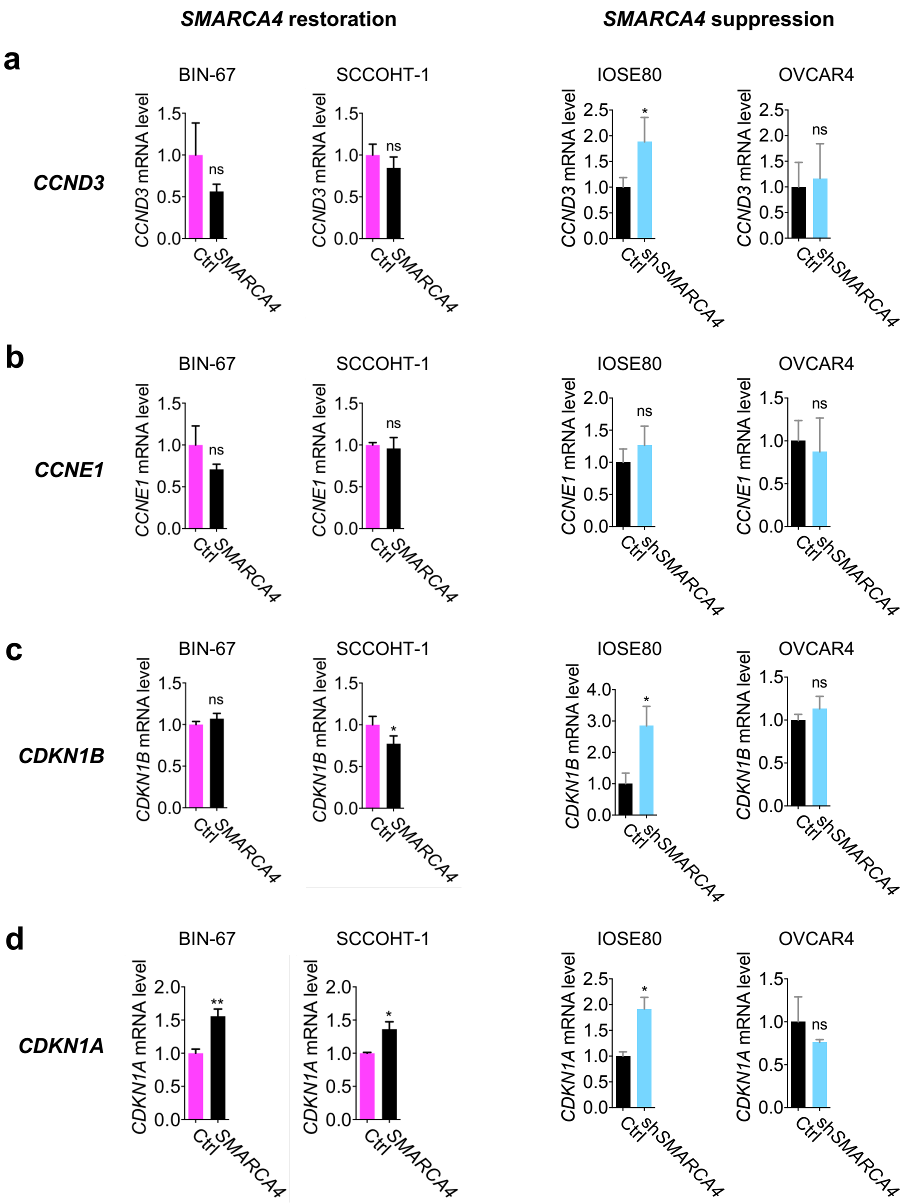

Supplementary Figure 8 | Expression of other relevant cell cycle genes in SCCOHT and control cells upon SMARCA4 perturbation.

Relative expression of *CCND3* (**a**), *CCNE1* (**b**), *CDKN1B* (**c**) and *CDKN1A* (**a**) mRNA (normalized to *GAPDH)* in BIN-67, SCCOHT-1, IOSE80 and OVCAR4 (before and after SMARCA4 perturbation) were measured by qRT-PCR. BIN-67/SCCOHT-1 expressing pReceiver or pReceiver-*SMARCA4* and IOSE80/OVCAR4 cells expressing pLKO control or sh*SMARCA4* were selected for 2 days for stable integration and then harvested for RNA. Error bars: mean ± s.d. of biological replicates (n=3; two-tailed *t*-test, * $p < 0.05$, ** $p < 0.01$; ns, not significant).

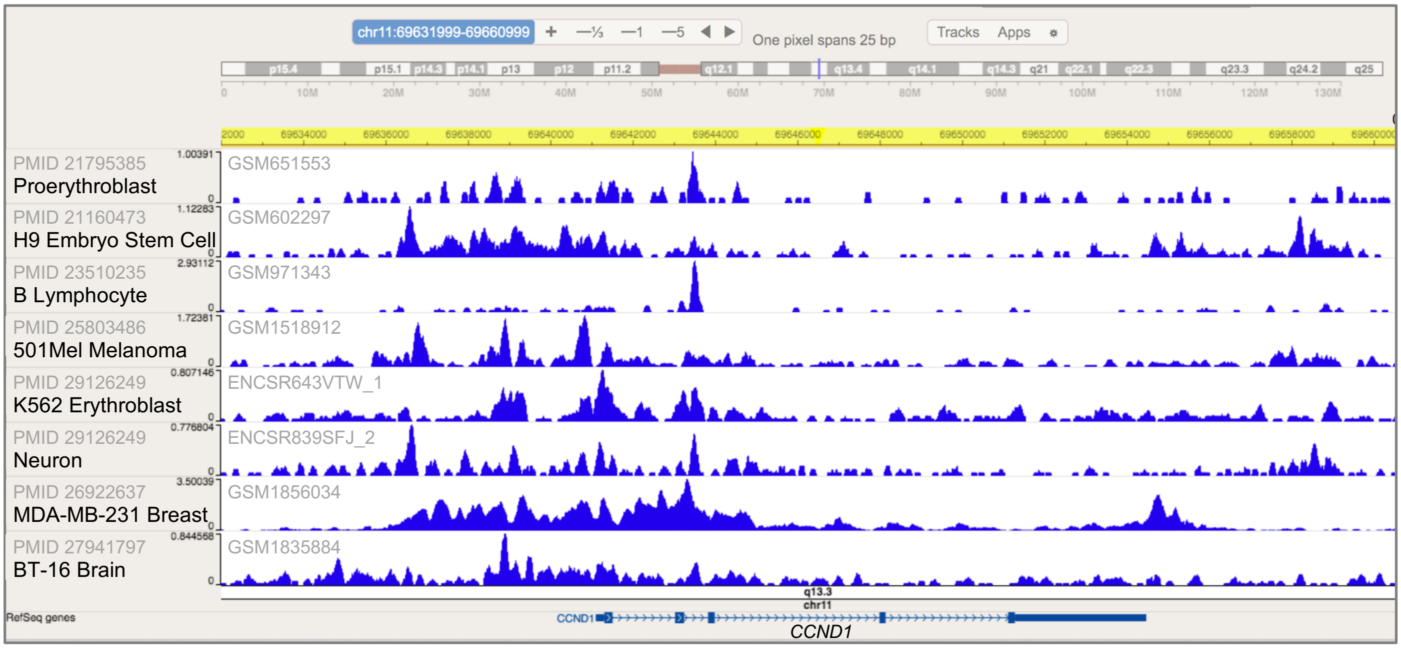

Supplementary Figure 9 | SMARCA4 occupancy at the *CCND1* promoter in additional human cell lines.

Publicly available ChIP-Seq tracks of SMARCA4 of 8 human cell lines of different tissue origins also show consistent SMARCA4 occupancy at the CCND1 promoter. Out of all SMARCA4 ChIP-Seq data sets available through Cistrome open source platform[1], there are 10 data sets of 8 different human cell lines that have fully passed quality controls as defined by Cistrome[1, 2, 3, 4, 5, 6, 7, 8]. Shown are the representative SMARCA4 ChIP-Seq tracks of these 8 cell lines visualized by Cistrome Data Browser - WashU Browser option (http://cistrome.org/db/#/). The corresponding PubMed ID (PMID) and data accession number (GEO or ENCODE) of these ChIP-Seq studies[2, 3, 4, 5, 6, 7, 8] are indicated.

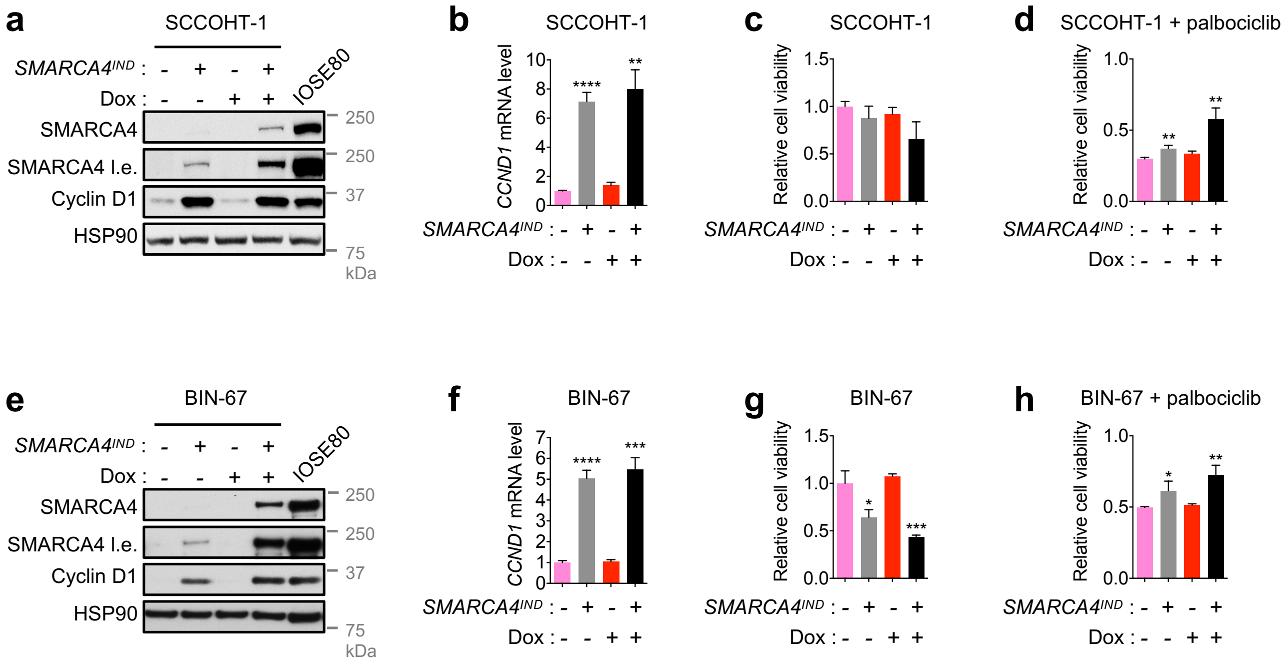

Supplementary Figure 10 | Low levels of SMARCA4-restoration with an inducible system in SCCOHT cells activate cyclin D1 expression and confers resistance to palbociclib.

a, **e,** Western blot analysis of indicated proteins in SCCOHT-1 (**a**) and BIN-67 (**e**) cells expressing pIN20 vector control or pIN20-*SMARCA4* cultured in the absence or presence of 5 ng/ml (SCCOHT-1) or 20 ng/ml (BIN-67) of doxycycline (Dox) for 2 days. IOSE80 was used as a control for comparison of SMARCA4 expression and HSP90 served as loading control.

b, **f**, Relative expression levels of *CCND1* mRNA (normalized to *GAPDH*) in these cells were measured by qRT-PCR. Error bars: mean ± s.d. of biological replicates (n=3; two-tailed *t*-test, ** $p < 0.01$, *** $p < 0.001$, **** $p < 0.0001$).

c, d, g, h, SCCOHT-1 and BIN-67 cells as described above were first cultured in the absence or presence of 5 ng/ml (SCCOHT-1) or 20 ng/ml (BIN-67) of doxycycline for 24 hours and followed by addition of 0 nM (**c, g**) or 250 nM (**d, h**) palbociclib treatment for 6 days. Cell viability was determined using CellTiter-Blue. (**c, g**) All groups were normalized to SCCOHT-1 or BIN-67 cells expressing the vector control without doxycycline. (**d, h**) Each group was normalized to its own control without palbociclib treatment. Error bars: mean ± standard deviation (s.d.) of biological replicates (n=3; two-tailed *t*-test, * $p < 0.05$, ** $p < 0.01$, *** $p < 0.001$).

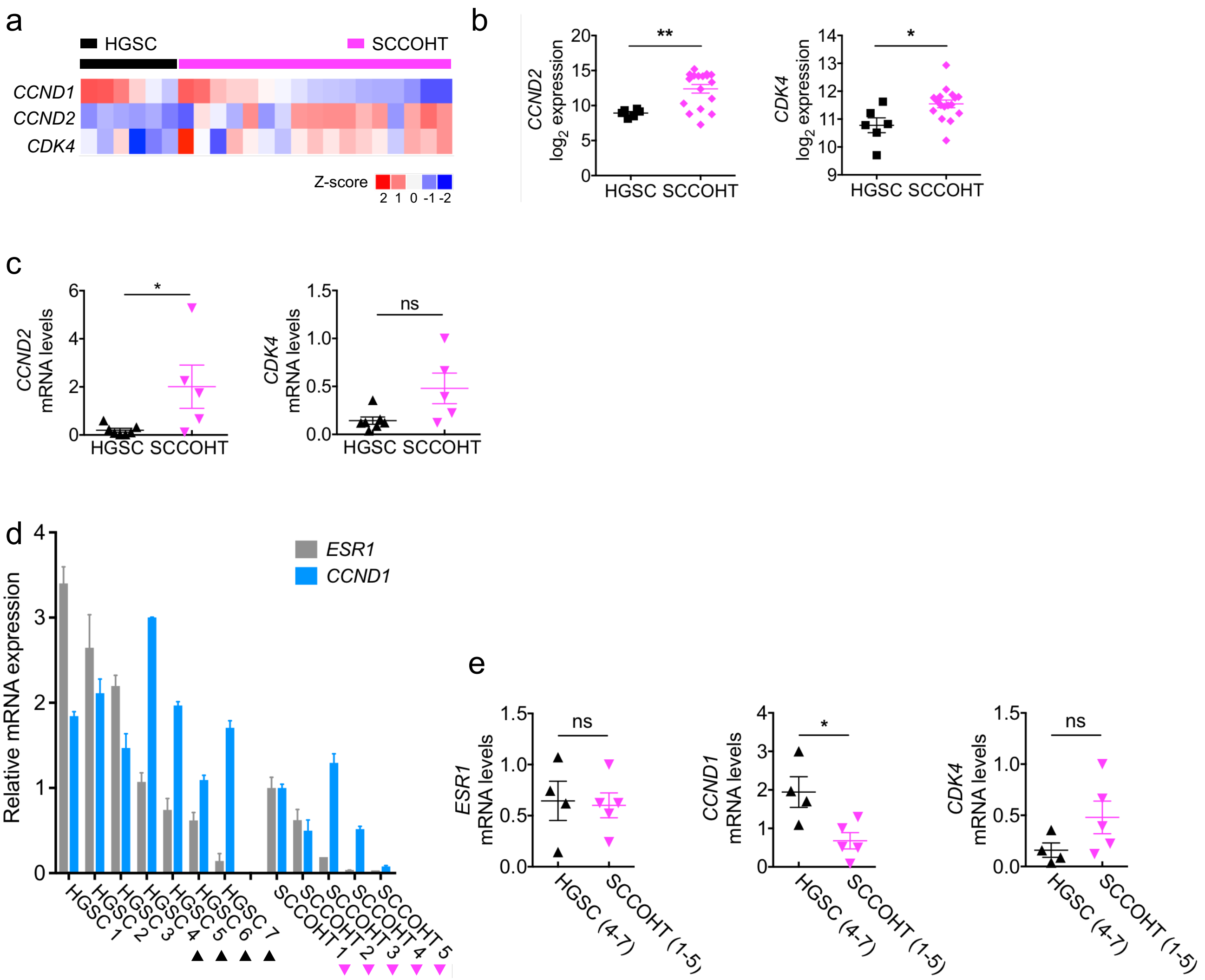

Supplementary Figure 11a-e | Expression analysis in SCCOHT and HGSC patient tumors

a, **b**, **c**, SCCOHT tumors express variable levels of *CCND2* and *CDK4* mRNA compared to ovarian high-grade serous carcinomas (HGSCs). Heatmaps (**a**) and boxplots (**b**) showing mRNA levels of indicated genes from a NanoString gene expression study in SCCOHT patient tumors (n = 17) relative to HGSCs (n = 6). Two-tailed *t*-test, * $p < 0.05$. (**c**) qRT–PCR analysis of an independent cohort of fresh-frozen patient tumor samples show that SCCOHT (n = 5) expressed variable levels of *CCND2* and *CDK4* mRNA (normalized to *GAPDH*) compared to HGSCs (n = 7). Error bars: t mean ± standard error of mean (s.e.m.); two-tailed *t*-test, ns, not significant.

d, **e**, SCCOHT patient tumors expressed reduced *CCND1* compared to HGSCs with similar *ESR1* expression. (**d**) Relative mRNA expression levels of *ESR1* and *CCND1* (normalized to *GAPDH*) in the HGSC (n=7) and SCCOHT (n=5) fresh-frozen patient tumor samples (same as Fig. 6c) were analyzed by qRT–PCR. (**e**) Comparison of *ESR1, CCND1* and *CDK4* mRNA expression in the HGSC (cases 4-7) and SCCOHT (cases 1-5) from above patient tumor samples. Error bars: mean ± standard error of mean (s.e.m.); two-tailed *t*-test, * $p < 0.05$; ns, not significant.

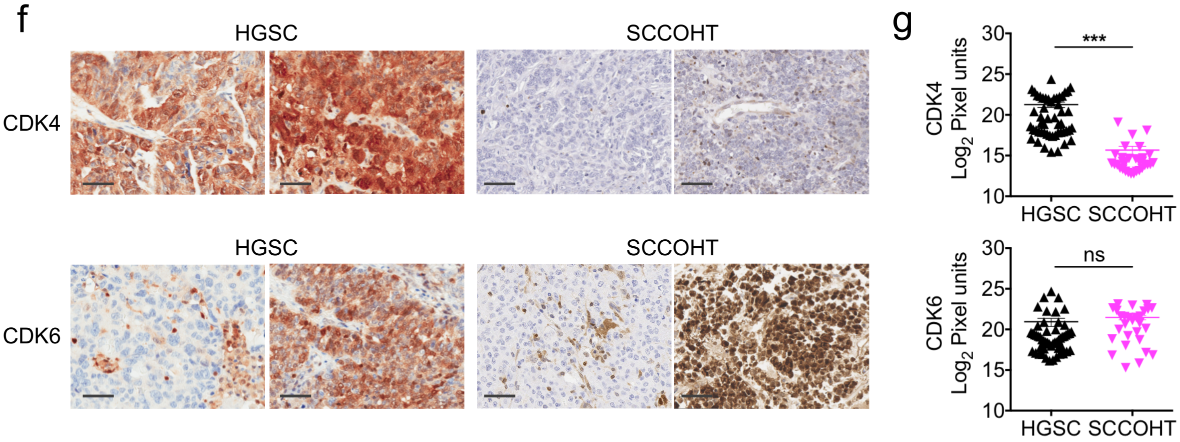

Supplementary Figure 11f, g | Expression analysis in SCCOHT and HGSC patient tumors

f, **g**, SCCOHT patient tumors express low levels of CDK4 and variable levels of CDK6 protein. Immunohistochemistry (IHC) analysis coupled with unbiased automated quantification[9] were performed on formalin-fixed paraffin embedded HGSC (n = 52) and SCCOHT (n = 32; 4 of which were also analyzed by qRT-PCR in **c**) patient tumors. Representative images of the IHC analysis (**f**) and quantification results (**g**) are shown. Error bars: mean ± standard error of mean (s.e.m.). Bar 50 μm; non-parametric Mann-Whitney test, *** $p < 0.001$; ns, not significant.

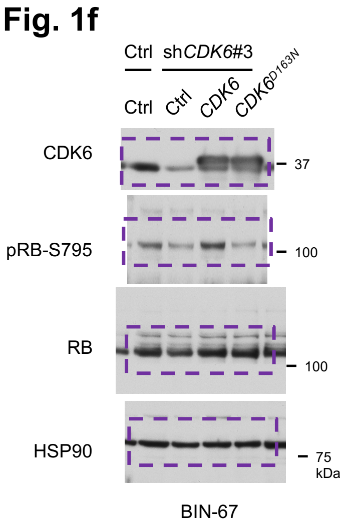

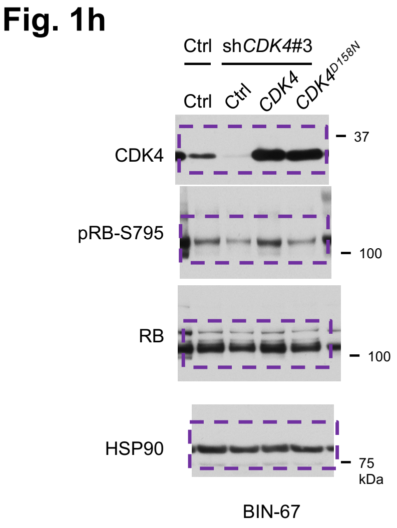

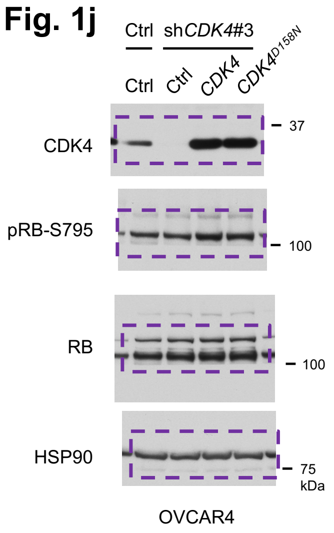

Supplementary Figure 12a | Uncropped scans for the key Western blots in Figure 1

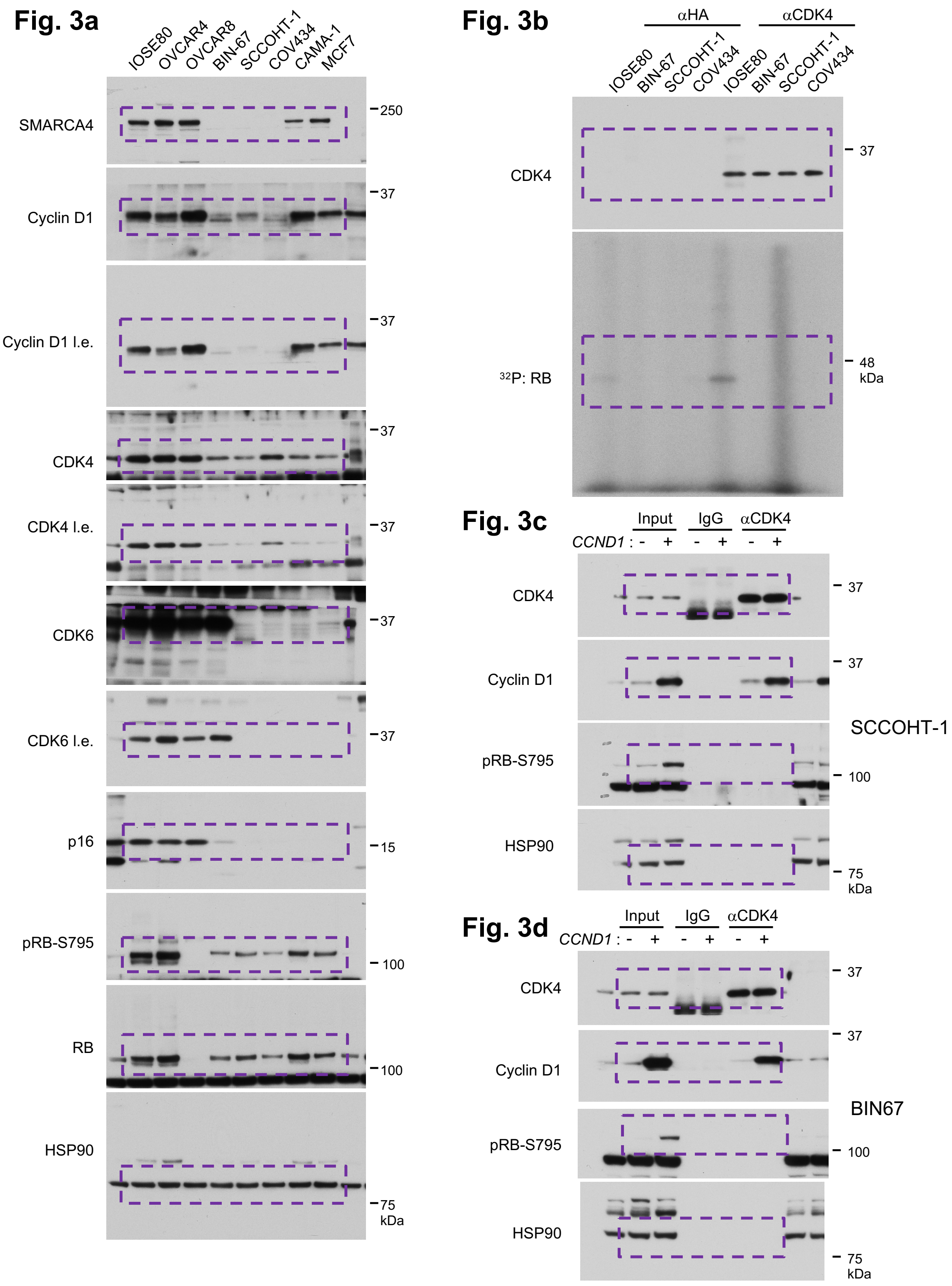

Supplementary Figure 12b | Uncropped scans for the key Western blots in Figure 3

Fig. 4e

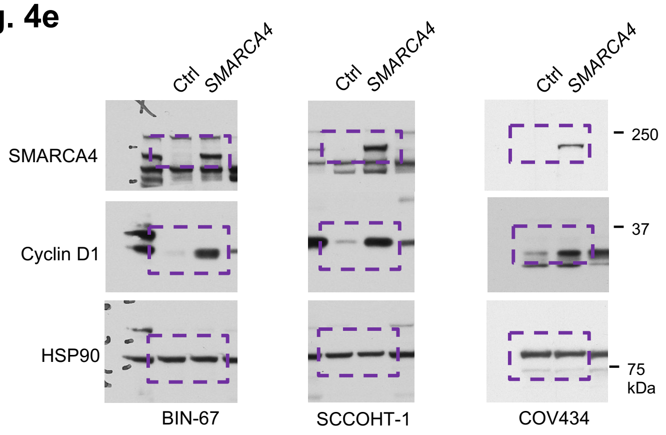

Fig. 4g

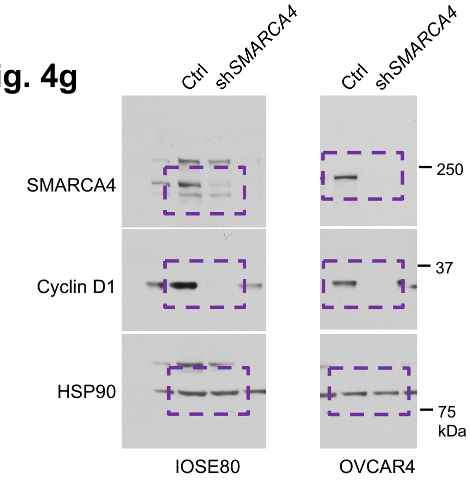

Supplementary Figure 12c | Uncropped scans for the key Western blots in Figure 4

Supplementary References

1. Liu T*, et al.* Cistrome: an integrative platform for transcriptional regulation studies. *Genome Biol* **12**, R83 (2011).

2. Wang X*, et al.* SMARCB1-mediated SWI/SNF complex function is essential for enhancer regulation. *Nat Genet* **49**, 289-295 (2017).

3. Takaku M*, et al.* GATA3-dependent cellular reprogramming requires activation-domain dependent recruitment of a chromatin remodeler. *Genome Biol* **17**, 36 (2016).

4. Rada-Iglesias A, Bajpai R, Swigut T, Brugmann SA, Flynn RA, Wysocka J. A unique chromatin signature uncovers early developmental enhancers in humans. *Nature* **470**, 279-283 (2011).

5. Laurette P*, et al.* Transcription factor MITF and remodeller BRG1 define chromatin organisation at regulatory elements in melanoma cells. *Elife* **4**, (2015).

6. Hu G*, et al.* Regulation of nucleosome landscape and transcription factor targeting at tissue-specific enhancers by BRG1. *Genome Res* **21**, 1650-1658 (2011).

7. Davis CA*, et al.* The Encyclopedia of DNA elements (ENCODE): data portal update. *Nucleic Acids Res* **46**, D794-D801 (2018).

8. Abraham BJ, Cui K, Tang Q, Zhao K. Dynamic regulation of epigenomic landscapes during hematopoiesis. *BMC Genomics* **14**, 193 (2013).

9. Venneti S*, et al.* Evaluation of histone 3 lysine 27 trimethylation (H3K27me3) and enhancer of Zest 2 (EZH2) in pediatric glial and glioneuronal tumors shows decreased H3K27me3 in H3F3A K27M mutant glioblastomas. *Brain Pathol* **23**, 558-564 (2013).
