# Supplementary material for: CDK4/6 inhibitors target SMARCA4-determined cyclin D1 deficiency in hypercalcemic small cell carcinoma of the ovary
Source: Nat Commun. 2019 Feb 4;10:558. doi: 10.1038/s41467-018-06958-9 (PMC6361890; doi:10.1038/s41467-018-06958-9)
Supplement: Supplementary file 3 — Description of Additional Supplementary Files [file 41467_2018_6958_MOESM3_ESM.pdf]

Description of Additional Supplementary Files

File Name: Supplementary Data 1

Description: Summary of the shRNA screen analysis using the MAGeCK statistical software package Listed are RRA values (robust rank aggregation), raw p-values (using permutation) and overall ranking of all genes screened in IOSE80, OVCAR4 and BIN-67 cells.

File Name: Supplementary Data 2

Description: Genes significantly regulated by SMARCA4 restoration in BIN-67 and SCCOHT-1 cells RNA-Seq was performed in BIN-67 and SCCOHT-1 cells with SMACRA4 restoration in triplicates. SMARCA4 target genes (fold change>3, adjusted p <0.05) in each cell line are listed.

File Name: Supplementary Data 3

Description: Summary of the pixel unit quantification of the HGSC and SCCOHT TMAs Listed are raw pixel units and the medians of cyclin D1, CDK4, CDK6, RB and p16 IHC signal in HGSC (n=52, Supplementary Data 3) and SCCOHT (n=32, Supplementary Data 4) embedded in tissue microarrays. Cores with low tumor cellularity and artifacts were not included in the analysis.
